# Primary autonomic failure: a complex case of orthostatic hypotension in a hypertensive elderly patient

**DOI:** 10.1093/ehjcr/ytae073

**Published:** 2024-02-05

**Authors:** Samah El-Mhadi, Najat Mouine, Halima Benjelloun, Souad Aboudrar, Mustapha El Bakkali

**Affiliations:** Cardiology A Department, Ibn Sina University Hospital Center, Rabat, Morocco; Department of Cardiology, Mohammed V Military Hospital, Rabat, Morocco; Cardiology A Department, Ibn Sina University Hospital Center, Rabat, Morocco; Exercise physiology and autonomic nervous system team, Laboratory of Physiology, Mohammed V University, Rabat, Morocco; Exercise physiology and autonomic nervous system team, Laboratory of Physiology, Mohammed V University, Rabat, Morocco

**Keywords:** Bradbury Eggleston syndrome, Primary autonomic failure, Autonomic nervous system, Supine arterial hypertension, Orthostatic arterial hypotension, Case report

## Abstract

**Background:**

Primary autonomic failure (PAF) or Bradbury Eggleston syndrome is a neurodegenerative disorder of the autonomic nervous system characterized by orthostatic hypotension.

**Case summary:**

We report the case of a 76-year-old patient with a history of hypertension, who presented with exercise-induced fatigue. He exhibited systolic hypertension and resting bradycardia in the supine position, with orthostatic hypotension without reactive tachycardia, suggesting dysautonomia. Neurological examination was unremarkable. The patient underwent cardiovascular autonomic testing, revealing evidence of beta-sympathetic deficiency associated with neurogenic orthostatic hypotension. Causes of secondary dysautonomia were excluded. The patient was diagnosed with PAF. Even if managing the combination of supine hypertension and orthostatic hypotension was challenging, significant improvements in functional and haemodynamic status were observed with a personalized management approach.

**Discussion:**

Throughout this case report, we emphasize the critical need for an evaluation of autonomic function and blood pressure’s dynamics in hypertensive patients experiencing orthostatic symptoms, enabling the implementation of tailored therapeutic strategies.

**Learning points**

- **Early Recognition of Dysautonomia:** The case emphasizes the importance of recognizing subtle signs of dysautonomia, such as exercise-induced fatigue and orthostatic hypotension. A comprehensive autonomic assessment can aid in early diagnosis and appropriate management of conditions like Pure Autonomic Failure.
- **Individualized Management Approach:** Managing the combination of supine hypertension and orthostatic hypotension requires an individualized approach. This case highlights the successful management achieved through a tailored strategy, combining medication adjustments, lifestyle modifications, and close monitoring. This underscores the significance of personalized care in optimizing patient outcomes.

## Introduction

Primary autonomic failure (PAF) is a neurodegenerative disorder of the autonomic nervous system characterized by orthostatic hypotension. The management of PAF is particularly challenging when it coexists with supine hypertension. This case report sheds light on the complexity of managing PAF in hypertension patients, aiming to improve tailored approaches to address this intricate medical scenario.

## Summary figure

**Table:** 

**Day 1:**	Presentation to the hospital with a history of arterial hypertension and recent exercise-induced fatigue. Physical examination: systolic hypertension in the supine position and orthostatic hypotension without reactive tachycardia, suggesting the presence of dysautonomia. Electrocardiogram: sinus bradycardia. Autonomic nervous system testing: evidence of vagal and beta-sympathetic deficiency associated with neurogenic orthostatic hypotension was assessed. Transthoracic echocardiography: moderate concentric left ventricular hypertrophy, no apex-base gradient, left ventricular ejection fraction: 61%, and global longitudinal strain: −20%
**Day 2 to 4:**	Further investigations: Bone scintigraphy, serum protein electrophoresis, serum and urine protein immunofixation and Bence Jones proteinuria, brain magnetic resonance imaging, and laboratory tests excluded secondary causes of dysautonomia.
**Day 5:**	Patient was diagnosed with primary autonomic failure. Management strategy: addressing supine hypertension and orthostatic hypotension with lifestyle modifications and medical prescriptions.
**Day 7:**	Discharge to home.
**After discharge:**	Weekly home Blood Pressure measurements were performed to closely monitor the patient's progress, resulting in significant improvements in functional and haemodynamic status.

## Case summary

We report the case of a 76-year-old male patient with a 6-year history of arterial hypertension on amlodipine at a dose of 10 mg once in the morning, who reported recent exercise-induced fatigue and presented to the Emergency department for further evaluation and management of his symptoms.

On admission, he exhibited symmetrical systolic arterial hypertension in the supine position (160/75 mmHg) and a resting heart rate of 50 bpm. When standing, he developed hypotension in the third minute, with BP dropping to 80/45 mmHg. No reactive tachycardia was observed, suggesting the presence of dysautonomia. The neurological examination revealed no signs of cerebellar, striatal, pyramidal, or extrapyramidal involvement; and there was no evidence of carpal tunnel syndrome.

The 12-lead electrocardiogram only showed sinus bradycardia.

**Figure 1:**
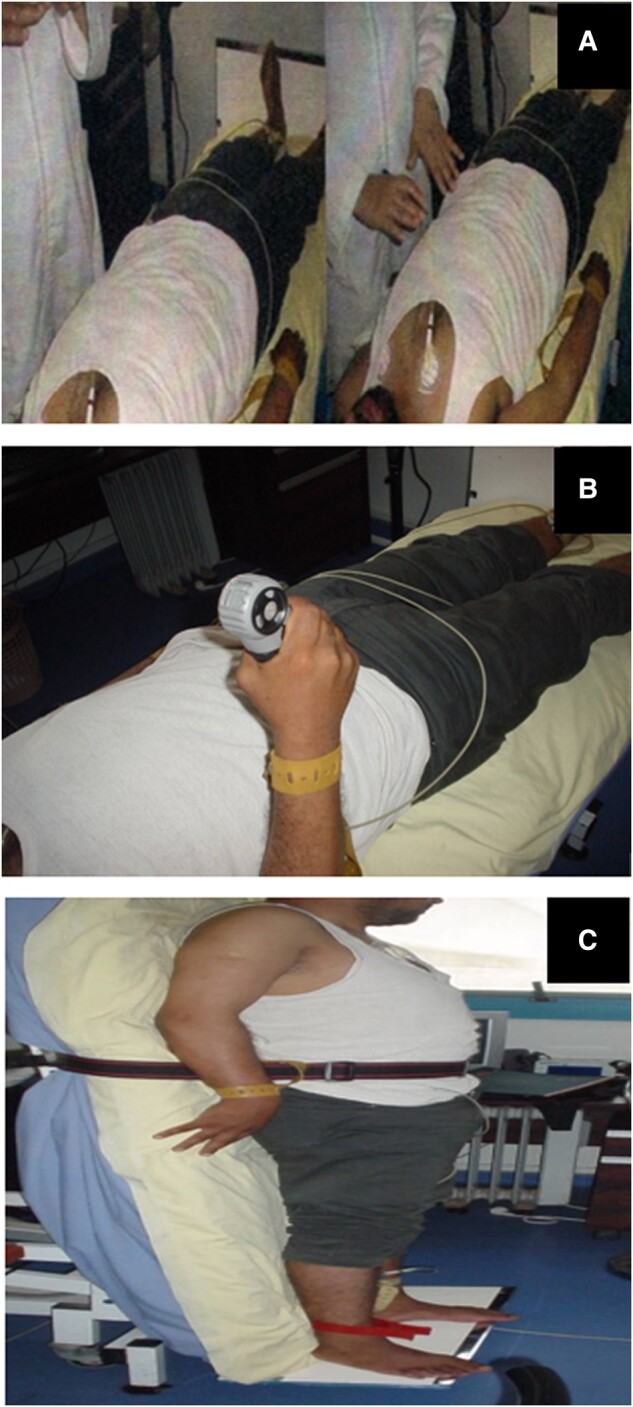
Cardiovascular autonomic tests: (*A*) deep breathing, (*B*) hand grip, and (*C*) tilt test.

The cardiovascular autonomic testing included deep breathing (DB), hand grip (HG), mental stress (MS), and tilt test (TT) (*Figure 1*). The results of the autonomic tests were as follows:

­ Vagal response obtained during the DB test was 12% (N: 30%)
­ Vagal response and alpha peripheral sympathetic response obtained on the hand grip test were 6% (N: 10%) and 20% (N: 10%), respectively
­ Alpha central sympathetic response and beta central sympathetic response obtained during MS test was 16% (N: 10%) and 3% (N: 10%), respectively
­ Vagal response, alpha peripheral adrenergic sympathetic response, and beta peripheral adrenergic sympathetic response obtained during the orthostatic test were 7% (N: 10%), 6% (N: 10%), and 7% (N: 10%), respectively.

Thus, there was evidence of vagal and beta-sympathetic deficiency associated with neurogenic orthostatic hypotension.

Further investigations were performed to exclude causes of secondary orthostatic hypotension. A transthoracic echocardiogram revealed moderate concentric left ventricular hypertrophy without systolic or diastolic dysfunction. The left ventricular ejection fraction was preserved at 61% and there was no apex-base gradient with a preserved longitudinal global strain at −20%.

Bone scintigraphy, serum protein electrophoresis, serum and urine protein immunofixation, and Bence Jones proteinuria screening ruled out cardiac senile transthyretin amyloidosis. Brain magnetic resonance imaging (MRI) ruled out central nervous system involvement.

Laboratory findings showed a normal complete blood count, electrolyte levels, and renal function. Morning cortisol levels, thyroid function tests, and glycated haemoglobin levels excluded metabolic disorders. Vitamin B9 and B12 levels were within target ranges. HIV serology was negative. Hence, the patient was diagnosed with PAF.

Managing the combination of supine hypertension and orthostatic hypotension was challenging for us. A 24-h ambulatory blood pressure monitoring (ABPM) was recommended before prescribing any medication to obtain an accurate assessment of the patient’s blood pressure (BP) profile.

To address supine hypertension, we switched from amlodipine to captopril as a short-acting antihypertensive medication; taken as a single evening dose of 25 mg/day before bedtime and only if systolic BP exceeds 140 mmHg. We also advised reducing sodium intake during evening meals and elevating the head of the bed while sleeping to enhance the nocturnal antihypertensive effect of captopril.

Conversely, for daytime orthostatic hypotension, we prescribed Etilefrine, an alpha and beta2 sympathomimetic amine, to be taken as a single morning dose of 20 mg; together with high-pressure compression stockings of 30 mmHg. We encouraged increasing salt and fluid intake (up to 3 g per day of sodium and 3 L per day of fluids); and avoiding hot environments. We also emphasized gradual postural changes.

**Figure 2:**
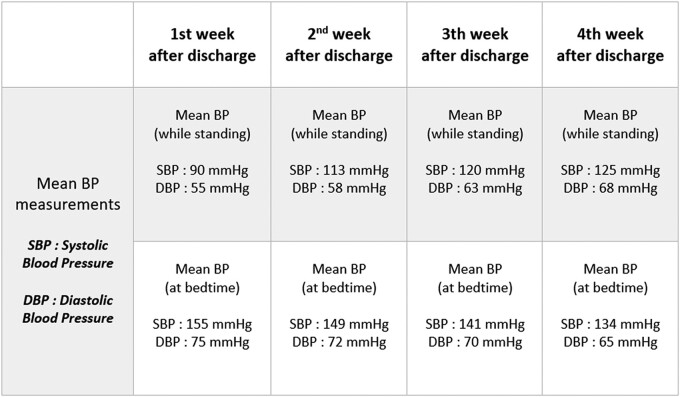
Summary table of weekly average blood pressure measurements after discharge.

Weekly home BP measurements were performed to closely monitor the patient's progress, resulting in significant improvements in functional and haemodynamic status over 6 months of follow-up (*Figure 2*).

## Discussion

Primary autonomic failure is a neurodegenerative disorder of the autonomic nervous system which was first described in 1925 by Bradbury and Eggleston.^1^

Nowadays, PAF is considered a α-synucleinopathy characterized by the deposition of α-synuclein in the ganglia and peripheral autonomic nerves, resulting in postganglionic autonomic failure. Peripheral sympathetic nerves are dysfunctional, leading to reduced production of catecholamines. Thus, inadequate sympathetic response to standing occurs due to peripheral cardiac and vasomotor denervation, contributing to orthostatic hypotension and other signs of autonomic dysfunction.^2^

The diagnosis of PAF follows the guidelines established in the consensus statement issued by the American Autonomic Society and the American Academy of Neurology in 1996.^3^ Orthostatic hypotension is the primary feature of PAF, but genitourinary, bowel, thermoregulatory, and haematological issues can also occur concurrently.

While the criteria specify the absence of neurologic features in PAF, there is growing awareness that PAF patients may develop other synucleinopathies such as multiple system atrophy, Parkinson’s disease, or dementia with Lewy bodies.^4^ To accurately distinguish PAF from other conditions presenting with orthostatic hypotension, a comprehensive clinical history, physical examination, and thorough neurological assessment should be conducted.

PAF typically presents in mid to late life and predominantly affects men. Orthostatic hypotension can present with or without symptoms and is defined as a sustained decrease in systolic blood pressure (SBP)of at least 20 mmHg and/or in diastolic blood bressure of at least 10 mmHg within 3 min of standing or tilting the head up by 60 degrees.^5^

Half of PAF patients experience coexisting supine hypertension due to impaired baroreflex function with increased sensitivity of adrenergic receptors and activation of mineralocorticoid receptors. When supine arterial hypertension is associated with orthostatic hypotension, a SBP reduction greater than 30 mmHg is required for diagnosis.^6^

Bedside testing for orthostatic blood pressure is useful for the initial diagnosis of orthostatic hypotension, but autonomic function testing especially the TT, is crucial for assessing its severity. Brain MRI is effective for excluding signs of central nervous system involvement.^7^

Laboratory findings may support the diagnosis of PAF when revealing low levels of supine norepinephrine with minimal to no increase upon standing; but their current clinical application requires further validation.^8^

While there is currently no cure for PAF, patients experience clinical improvement through non-pharmacological and pharmacological interventions aimed at controlling BP fluctuations.

Adjusting medications that contribute to orthostatic hypotension and patients’ education on lifestyle modifications such as gradual position changes and avoiding hot environments, are fundamental. Non-pharmacological measures focus on increasing fluid and salt intake and wearing venous compression stockings during orthostatism. Elevating the head of the bed and decreasing fluid and salt intake is recommended at bedtime to alleviate supine hypertension.

Pharmacological interventions for orthostatic hypotension should be used in conjunction with non-pharmacological approaches. Midodrine, droxidopa, pyridostigmine, and fludrocortisone are Food and Drug Adinistration-approved for treating orthostatic hypotension.^9^

As for supine hypertension, transdermal agents like nitroglycerine or clonidine and oral agents with short duration of action such as captopril, nifedipine, losartan, or nebivolol can be prescribed.^10^

Additionally to blood pressure management, a multidisciplinary approach involving interventions such as physical and cardiorespiratory rehabilitation programs and psychological support has been shown to enhance patients’ quality of life.

## Conclusion

This case report emphasizes the critical need for a comprehensive evaluation of autonomic function and BP dynamics in hypertensive patients experiencing orthostatic symptoms, enabling the implementation of tailored therapeutic strategies.

## Data Availability

No new data were generated or analyzed in support of this article.

## Lead author biography

Doctor EL-MHADI SAMAH, I am a resident in the field of Cardiology and Vascular Diseases at Ibn Sina University Hospital in Rabat, Morocco.
